# Supplementary material for: A Role for Cytosolic Fumarate Hydratase in Urea Cycle Metabolism and Renal Neoplasia
Source: Cell Rep. 2013 May 30;3(5):1440–8. doi: 10.1016/j.celrep.2013.04.006 (PMC3675675; doi:10.1016/j.celrep.2013.04.006)
Supplement: Document S1. Article plus Supplemental Information [file mmc2.pdf]

Cell PRESS
Open ACCESS

Cell Reports
Report

A Role for Cytosolic Fumarate Hydratase in Urea Cycle Metabolism and Renal Neoplasia

Julie Adam,[1] Ming Yang,[1,14] Christina Bauerschmidt,[1,14] Mitsuhiro Kitagawa,[4,14] Linda O'Flaherty,[1,14] Pratheesh Maheswaran,[1] Gizem Özkan,[1] Natasha Sahgal,[6] Dilair Baban,[7] Keiko Kato,[4] Kaori Saito,[4] Keiko Iino,[4] Kaori Igarashi,[4] Michael Stratford,[9] Christopher Pugh,[2] Daniel A. Tennant,[10] Christian Ludwig,[10] Benjamin Davies,[8] Peter J. Ratcliffe,[2] Mona El-Bahrawy,[11,12] Houman Ashrafian,[13] Tomoyoshi Soga,[4,5] and Patrick J. Pollard[1,3,*]

[1]Cancer Biology and Metabolism Group
[2]Hypoxia Biology Group
[3]Oxford-Keio Metabolomics Consortium
Nuffield Department of Medicine, Henry Wellcome Building for Molecular Physiology, University of Oxford, Oxford OX3 7BN, UK
[4]Institute for Advanced Biosciences
[5]Oxford-Keio Metabolomics Consortium
Keio University, 246-2 Mizukami, Tsuruoka, Yamagata 997-0052, Japan
[6]Bioinformatics and Statistical Genetics
[7]High Throughput Genomics
[8]Transgenic Core
Wellcome Trust Centre for Human Genetics, University of Oxford, Oxford OX3 7BN, UK
[9]Gray Institute for Radiation Oncology and Biology, Department of Oncology, Old Road Campus Research Building, University of Oxford, Roosevelt Drive, Oxford OX3 7DQ, UK
[10]School of Cancer Sciences, College of Medical and Dental Sciences, University of Birmingham, Edgbaston, Birmingham B15 2TT, UK
[11]Department of Histopathology, Imperial College London, Hammersmith Hospital, London W12 0NN, UK
[12]Department of Pathology, Faculty of Medicine, University of Alexandria, Alexandria, Egypt
[13]Department of Cardiovascular Medicine, University of Oxford, Oxford OX3 9DU, UK
[14]These authors contributed equally to this work
*Correspondence: paddy@well.ox.ac.uk
http://dx.doi.org/10.1016/j.celrep.2013.04.006

SUMMARY

The identification of mutated metabolic enzymes in hereditary cancer syndromes has established a direct link between metabolic dysregulation and cancer. Mutations in the Krebs cycle enzyme, fumarate hydratase (FH), predispose affected individuals to leiomyomas, renal cysts, and cancers, though the respective pathogenic roles of mitochondrial and cytosolic FH isoforms remain undefined. On the basis of comprehensive metabolomic analyses, we demonstrate that FH1-deficient cells and tissues exhibit defects in the urea cycle/arginine metabolism. Remarkably, transgenic re-expression of cytosolic FH ameliorated both renal cyst development and urea cycle defects associated with renal-specific FH1 deletion in mice. Furthermore, acute arginine depletion significantly reduced the viability of FH1-deficient cells in comparison to controls. Our findings highlight the importance of extramitochondrial metabolic pathways in FH-associated oncogenesis and the urea cycle/arginine metabolism as a potential therapeutic target.

INTRODUCTION

Since first highlighted in the last century, altered metabolism has been a consistent observation in cancer cells (Warburg, 1956). Recently, the identification of mutated Krebs cycle enzymes in familial cancer syndromes has linked altered metabolism and cancer directly (reviewed in Bayley and Devilee, 2010; Frezza et al., 2011a). Mutations in one of these enzymes, fumarate hydratase (FH), predispose individuals to hereditary leiomyomatosis and renal cell cancer (HLRCC) (Tomlinson et al., 2002). Affected individuals also develop renal cysts, a phenotype that is recapitulated in FH1 (murine FH)-deficient mice (Pollard et al., 2007). Loss of FH activity results in accumulation of intracellular fumarate, which, in turn, affects multiple signaling pathways, including inhibition of 2-oxoglutarate (2OG)-dependent dioxygenase enzymes (Isaacs et al., 2005; Loenarz and Schofield, 2008; O'Flaherty et al., 2010; Pollard et al., 2005; Xiao et al., 2012) and posttranslational modification (succination) of cysteine residues (Adam et al., 2011; Alderson et al., 2006; Bardella et al., 2011; Yang et al., 2012). However, the mechanism(s) of tumorigenesis and particularly the role of defective mitochondrial metabolism in FH-associated disease remain undetermined. Though considered a Krebs cycle enzyme, FH is also expressed in the cytosol and the nucleus (Yogev et al., 2010, 2011). Moreover, re-expression of cytosolic FH ameliorates constitutive activation of both the hypoxia and antioxidant response pathways in FH1-null cells, despite a persistent defect in oxidative metabolism (Adam et al., 2011; O'Flaherty et al., 2010). To investigate the role of extramitochondrial FH in renal cyst development, we have undertaken high-resolution mass-spectrometry-based metabolomic analyses of FH-deficient cells, renal cysts, and tumors. To corroborate our findings in vivo, we generated two transgenic murine models where either FH or extramitochondrial FH (FH^{cyt}) is stably expressed from the

Rosa26 locus (Zambrowicz et al., 1997). We demonstrate that re-expression of cytosolic FH in FH1-deficient mice is critical for the suppression of renal cyst development and restoration of defects in the arginine biosynthesis pathway. Furthermore, FH-deficient cells exhibit a greater dependence on exogenous arginine than wild-type counterparts. Taken together, our data support a role for extramitochondrial metabolic pathways in renal neoplasia and arginine deprivation as a candidate target for therapy.

RESULTS

Urea Cycle Metabolites Accumulate in FH1KO Kidneys

Previously, we demonstrated that mice with deletion of FH1 in renal tubular epithelial cells (Shao et al., 2002) (FH1$^{flox/flox}$ Ksp-Cre$^{+/-}$; FH1KO) develop hyperplastic renal cysts (Pollard et al., 2007). This model has been characterized further by genetic crosses and subsequent gene expression analyses (Adam et al., 2011; Ashrafian et al., 2010), but without comprehensive analysis of metabolism. Therefore, we determined metabolite levels in control and FH1KO kidneys using capillary electrophoresis time-of-flight mass spectrometry (CE-TOFMS; Soga et al., 2009). Levels of fumarate, argininosuccinate, and citrulline were increased significantly in FH1KO kidneys compared to controls, whereas aspartate was depleted (Figures 1A–1D; Table S1). Metabolic pathway analyses using IPA (Ingenuity Pathway Analysis, Ingenuity Systems) showed significant changes in the urea cycle/arginine biosynthesis pathway (Table S1).

FH1KO Mouse Embryonic Fibroblasts Exhibit Multiple Defects in the Krebs Cycle and Utilize the Urea Cycle, but Not Reductive Carboxylation

There were at least two hypotheses to test: whether the urea cycle is dysregulated in the FH1KO mouse embryonic fibroblasts (MEFs) as predicted above, and whether they use the reductive carboxylation pathway as has been reported for other FH-deficient cells (Mullen et al., 2012). Hence, we cultured wild-type (FH1WT) and FH1KO MEFs in medium containing the stable isotope tracer glutamine-2,3,3,4,4-d5 ([D5]-glutamine) for 3 and 9 hr and determined the incorporation of deuterium label in Krebs cycle and urea cycle metabolites by CE-TOFMS analyses (Figure 1E; Table S1). Use of [D5]-glutamine by the canonical oxidative Krebs cycle would result in m+4 for 2OG and succinate, m+2 for fumarate and malate, and m+1 for oxaloacetate and aspartate and thus provides a means of differentiating whether argininosuccinate is generated by arginine and fumarate, or alternatively by condensation of citrulline and aspartate (Figure 1E). Significantly, we detected argininosuccinate m+2, and, in addition, the isotopic distribution pattern of argininosuccinate matched that of fumarate, but not of aspartate (Figure 1E). Therefore, we concluded that argininosuccinate is synthesized directly from fumarate. The glutamine-dependent reductive carboxylation pathway metabolizes 2OG to citrate for lipid synthesis, forcing partial reversal of the Krebs cycle (Metallo et al., 2012; Mullen et al., 2012; Wise et al., 2011). We did not observe evidence for a reversal of the Krebs cycle in our MEF model, and furthermore, levels of citrate, *cis*-aconitate, and isocitrate were significantly lower in FH1KO compared to FH1WT MEFs (Figure 1E; Table S1). We have proposed previously that, in FH1KO MEFs, 2OG can be converted to isocitrate by isocitrate dehydrogenase (IDH), but isocitrate cannot be further metabolized to citrate, probably as a result of impaired Aconitase 2 activity due to succination. We did not observe any label in citrate and have suggested that this may be the consequence of inactive aconitase in both the mitochondria and cytoplasm of FH1KO MEFs (Ternette et al., 2013).

Cytosolic FH Suppresses Renal Cyst Development In Vivo

Given that the part of the urea cycle affected by fumarate accumulation functions in the cytosol (Shambaugh, 1977), we hypothesized that cytosolic FH may be important in the pathogenesis of HLRCC. Previously, we demonstrated that expression of cytosolic FH in FH1KO MEFs reduced fumarate levels significantly with concomitant loss of nuclear factor (erythroid-derived 2)-like 2 (NFE2L2/NRF2) and hypoxia-inducible factor (HIF) expression, but did not restore defects in oxidative metabolism (Adam et al., 2011; O’Flaherty et al., 2010). To investigate the in vivo role of cytosolic FH, we constructed two transgenic mouse lines stably expressing either FH or FH^{cyt} (excluded from the mitochondria) with a C-terminal V5 affinity tag and under the control of the CAG promoter (Niwa et al., 1991). Equivalent expression between both lines was ensured by targeting the FH transgenes to the Rosa26 locus (Zambrowicz et al., 1997) using integrase-mediated cassette exchange (Chen et al., 2011) (Figures 2A and S1). Targeting fidelity was assessed using PCR (Figure S1), and FH protein localization was confirmed in embryonic stem (ES) cells by immunofluorescence (Figures 2B and 2C). Transgenic expression of FH-V5 was analyzed by immunoblotting and immunofluorescence (Figure S1). Similar to HLRCC patients with renal cancer, mice with kidney-specific FH1 deletion develop hyperplastic renal cysts (Pollard et al., 2007). We intercrossed FH1KO mice with both transgenic lines (FH1KO+FH and FH1KO+FH^{cyt}). Macroscopic analyses of kidneys from 30-week-old mice (Figure 2D) indicated that expression of either transgene was sufficient to ameliorate the increased renal mass in FH1KO mice, and microscopic analysis at three time points (13, 20, and 30 weeks) confirmed that transgenic expression of cytosolic FH was sufficient to suppress cyst development (Figures 2E–2H).

Cytosolic Expression of FH in FH1KO Mice Restores Urea Cycle Metabolism

Since re-expression of cytosolic FH “rescued” the cystic phenotype associated with FH1 deletion, we hypothesized that this might be mediated in part through alterations in metabolism. Hence, we determined the metabolic consequences of restoring cytosolic FH in FH1KO kidneys from 15-week-old mice by CE-TOFMS and IPA analysis (Table S1). This time point was chosen to avoid severe pathological changes in the kidneys and to match previous analyses (Adam et al., 2011). Metabolites showing the most significant changes are indicated using a heatmap (Figure 3A). Notably, transgenic rescue of FH1 deficiency with either FH or FH^{cyt} restored levels of fumarate and urea cycle

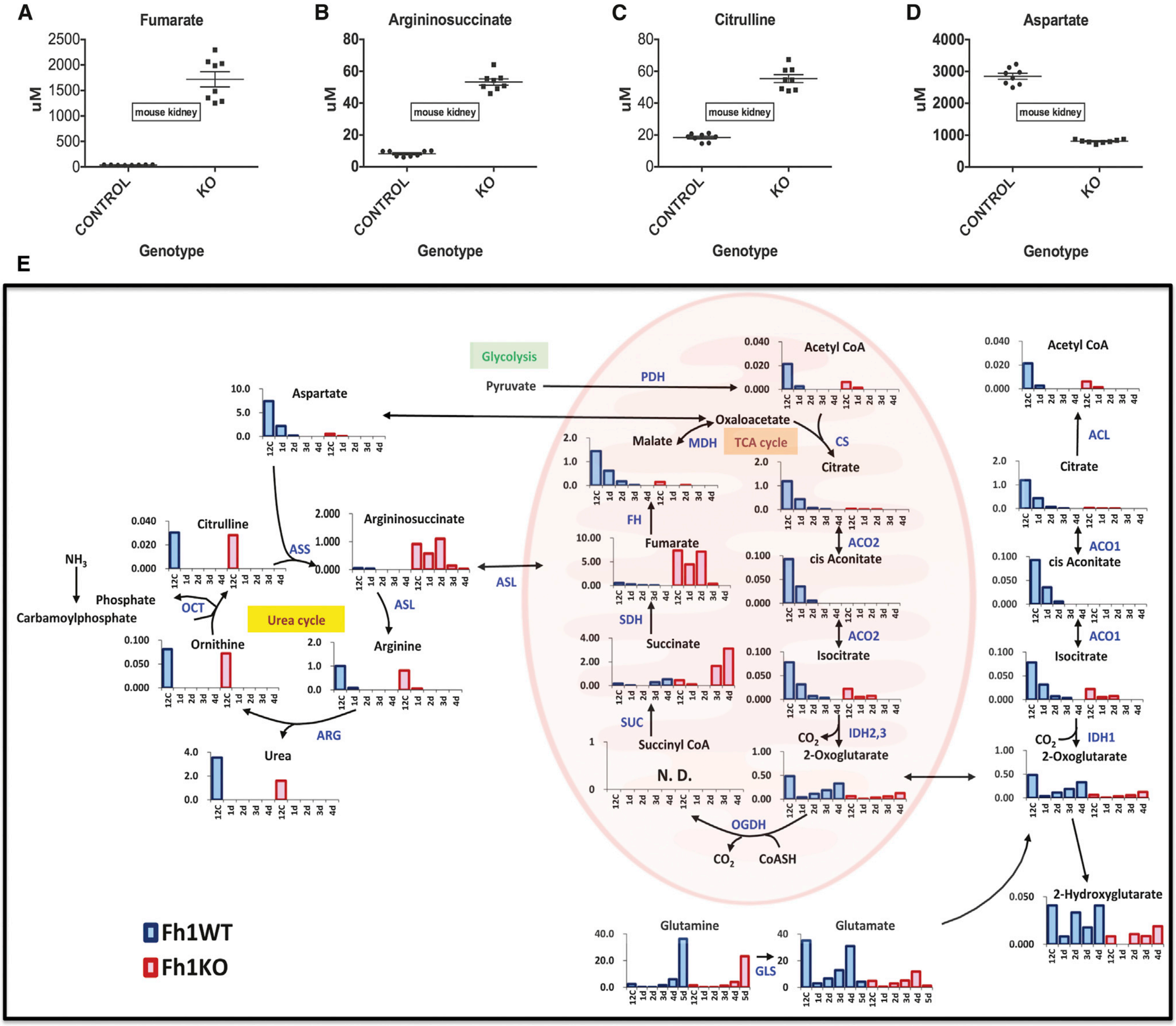

Figure 1. FH-Deficient Cells Synthesize Argininosuccinate Directly from Fumarate

(A–D) Concentrations of specific urea cycle metabolites (μM) in control and FH1KO kidneys as determined by CE-TOFMS (Soga et al., 2009). All differences between control and FH1KO mice were significant ($p < 0.01$, Student's t test). For metabolomic analyses, six mice aged 15 weeks were analyzed from each group.

(E) CE-TOFMS analyses of deuterium label incorporation into key Krebs cycle and urea cycle metabolites in FH1WT (blue) and KO (red) MEFs after 9 hr incubation in culture containing [D5]-glutamine. Transit of label through the canonical oxidative Krebs cycle would result in 2OG+4, succinate+4, fumarate+2, malate+2, and Asp+1, while reductive carboxylation of glutamate would result in isocitrate m+2, citrate m+2, and aspartate m+1. We did not observe label enrichment in citrate, so the reductive mechanism is not used for citrate synthesis. Argininosuccinate produced from arginine and fumarate has m+2, whereas that produced from citrulline and aspartate has m+1. We detected predominantly argininosuccinate m+2, which has a similar isotopomer distribution pattern to fumarate, suggesting it is synthesized directly from fumarate.

For each graph, the concentration of metabolites (fmol/cell) is indicated on the y axis and label enrichment of [D5]-glutamine in FhWT and KO MEFs are represented on the x axis in the following order: 12C, 12C-1d, 12C-2d, 12C-3d, and 12C-4d. See also Table S1 for absolute metabolite levels.

ACL, ATP citrate lyase; ACO1, -2, aconitase 1, -2; IDH1, -2, -3, isocitrate dehydrogenase 1, 2, 3; CS, citrate synthase; SUC, succinyl CoA synthetase; SDH, succinate dehydrogenase; OGDH, oxoglutarate dehydrogenase; FH, fumarate hydratase; MDH, malate dehydrogenase; PDH, pyruvate dehydrogenase; GLS, glutaminase; ASS, argininosuccinate synthase; ASL, argininosuccinate lyase; OCT, ornithine carbamoyltransferase; ARG, arginase.

metabolites comparable to controls (Figures 3B–3E). Interestingly, levels of citrulline (which exists in both the mitochondria and cytosol) were not fully rescued in FH1KO+FH^{cyt} animals (Figure 3E).

Fumarate and Argininosuccinate Levels Are Increased in HLRCC Tumors

To determine whether the increase in urea cycle metabolites observed in FH1KO kidneys was recapitulated in FH mutant

Cell PRESS
Open ACCESS

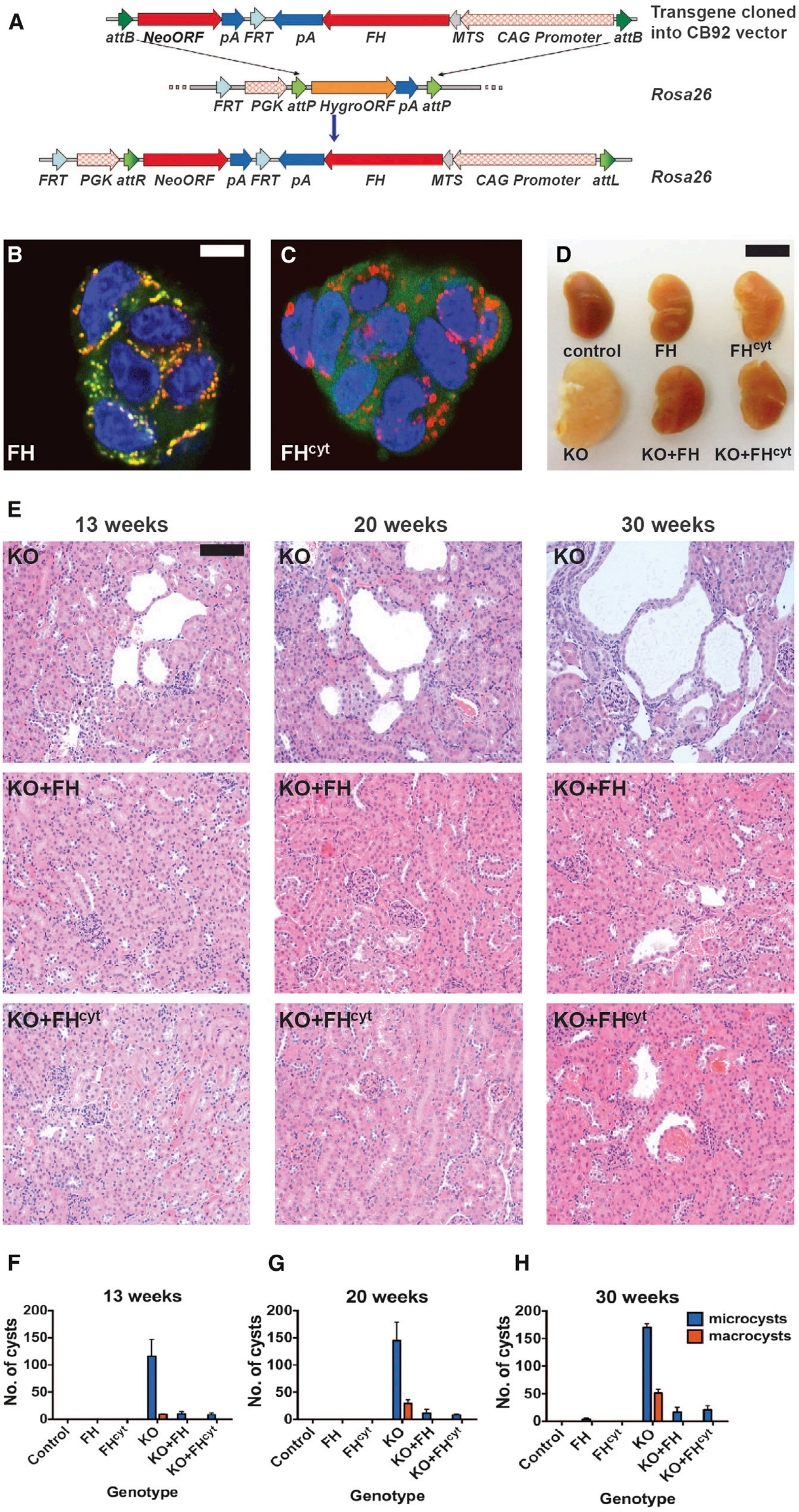

Figure 2. Generation and Analyses of FH-Expressing Transgenic Mice to Investigate the Role of Cytosolic FH/Fumarate in Renal Cyst Development

(A) The FH and FH^{cyt} transgenes were cloned into the CB92 vector and targeted to the *Rosa26* locus, using phage-mediated recombination (Chen et al., 2011).

(B and C) Localization of FH and FH^{cyt} was confirmed in ES cells by immunocytochemistry. Colocalization (yellow) of FH (green) and mitochondria (red) is evident (B), whereas FH^{cyt} is absent from the mitochondria (C). Nuclei (blue) are visualized with DAPI.

(D) Kidneys harvested from 30-week-old control, transgene-only (FH, FH^{cyt}), and genetically "rescued" mice (FH1KO+FH and FH1KO+FH^{cyt}) appeared macroscopically normal compared to FH1KO kidneys, which appeared enlarged and cystic.

(E) Hematoxylin and eosin (H&E) staining of kidneys harvested from mice at 13, 20, and 30 weeks reveal that transgenic expression of either FH or FH^{cyt} is sufficient to ameliorate cyst development.

(F–H) Numbers and frequency of macrocysts (>0.5 mm) and microcysts (>0.1 mm) were determined in each group (n = 6). Error bars represent the SEM.

Scale bars, 5 μm (B and C), 10 mm (D), and 100 μm (E). Error bars indicate SEM. See also Figure S1.

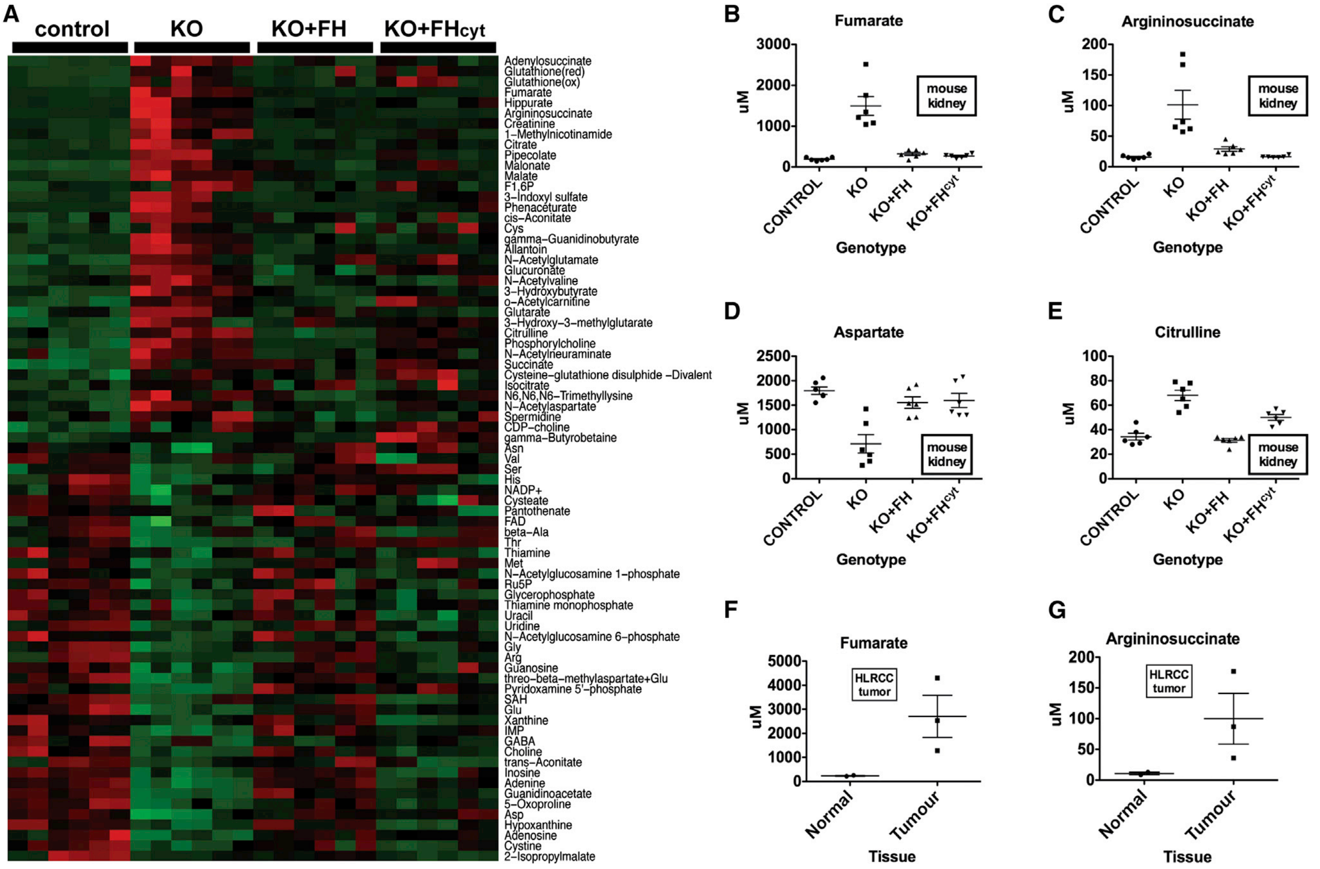

Figure 3. Metabolite Analysis of Kidneys from FH1KO- and FH-Reconstituted Mice

(A) Heatmap showing relative levels of specific metabolites in kidneys from control, FH1 KO, and reconstituted (FH1 KO+FH and FH1 KO+FH^{cyt}) mice. For each group, kidneys from six mice (aged 15 weeks) were analyzed using CE-TOFMS (Soga et al., 2009), and the data were filtered to select only metabolites where there was a 2-fold difference between any group and a p value <0.05 (one-way ANOVA). Relative metabolite levels are indicated by red (high) and green (low).

(B–E) Concentrations (μM) of the specific urea cycle metabolites fumarate (B), argininosuccinate (C), aspartate (D), and citrulline (E) measured in kidneys from mice of each of the above four genotypes.

(F and G) Analysis of FH mutant renal cancers (n = 3) and adjacent normal tissue (n = 2) indicates accumulation of fumarate (F) and aspartate (G).

Error bars indicate SEM. See also Table S1.

tumors, we used CE-TOFMS and IPA to analyze the metabolome of normal kidney (n = 2) and type 2 papillary tumors (n = 3) from two HLRCC patients (Table S1). Increased levels of both fumarate and argininosuccinate were observed in the tumors (Figures 3F and 3G).

Argininosuccinate Is Generated in FH-Deficient Cells via Reversal of Argininosuccinate Lyase

Argininosuccinate can be generated either from citrulline and aspartate (through argininosuccinate synthetase [ASS1]) or from fumarate and arginine (through reversal of argininosuccinate lyase [ASL]) (Figure 1E; Wheatley, 2005). To confirm independently the route by which argininosuccinate accumulates in FH1 deficiency, we cultured WT and FH1KO MEFs in medium containing uniformly labeled [U-$^{15}N_4$, U-$^{13}C_6$] arginine and analyzed the cells using heteronuclear single quantum correlation nuclear magnetic resonance (HSQC-NMR) (Ludwig and Günther, 2011). We observed labeled argininosuccinate in FH1KO cells but not in WT cells (Figure 4A), consistent with a reversed ASL reaction in these cells. We then analyzed two panels of isogenic FH-deficient lines; FH1KOMEFs reconstituted with either FH or FH^{cyt} (O'Flaherty et al., 2010) and UOK262 (derived from a metastasis from an FH mutant renal cancer (Yang et al., 2010b), which we have genetically complemented with the same FH or FH^{cyt} constructs (Figure S2). CE-TOFMS analysis demonstrated that re-expression of either FH- or FH^{cyt}-suppressed fumarate (Figure 4B) and argininosuccinate (Figure 4C) accumulation in both FH-deficient cell lines. We observed argininosuccinate m+10 as the predominant isotopomer (Figure 4C), indicating the direct formation of argininosuccinate from arginine and fumarate, thus corroborating the data from the [D5]-glutamine labeling (Figure 1E).

FH-Deficient Cells Exhibit Increased Sensitivity to Arginine Deprivation

Recent studies have demonstrated that depletion of arginine in ASS1-negative tumors inhibits their growth and predicts clinical benefit (Feun et al., 2012; Kelly et al., 2012; Yang et al., 2010a).

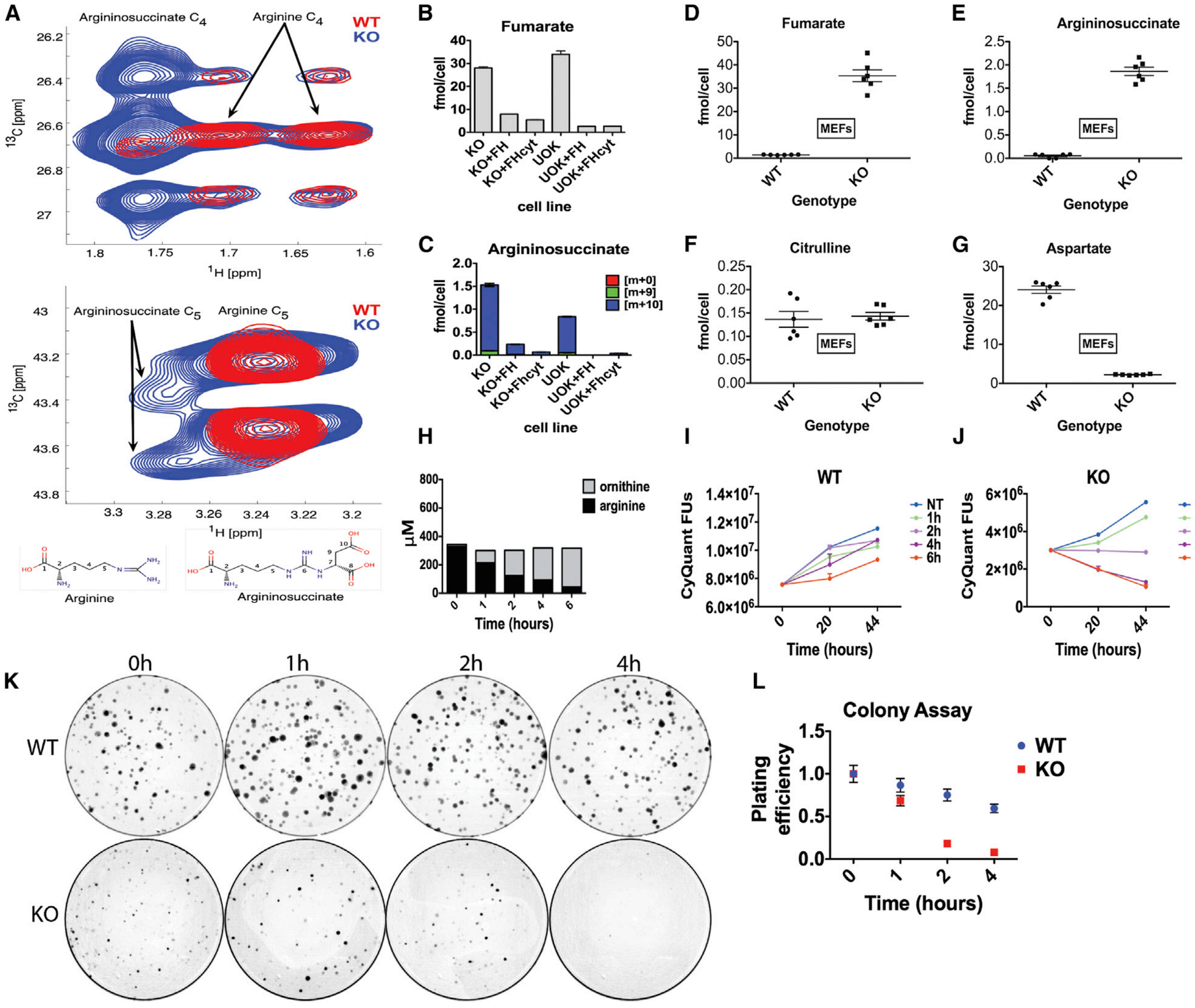

Figure 4. Argininosuccinate Is Produced from Arginine in FH-Deficient Cells

(A) Sections of HSQC spectra for the C_4 (top panel) and C_5 (bottom panel) resonances of arginine and argininosuccinate detected in FH1WT (red) and FH1KO (blue) MEFs. Argininosuccinate was detected in FH1KO MEFs only, which also correlates with the appearance of the fumarate resonance at 6.5/138 ppm.

(B and C) Cells of the indicated genotype were cultured in media containing $^{15}N_4$,$^{13}C_6$-arginine for 12 hr prior to metabolite extraction and analyses of fumarate (B) and argininosuccinate (C) levels by CE-TOFMS (Soga et al., 2009). The m+10 isotopomer comprises >90% of total argininosuccinate detected in FH1KO MEFs and UOK262 cells, suggesting direct conversion of argininosuccinate from arginine and fumarate. Three independent cultures were used for each condition, and data shown here are representative of two independent experiments.

(D–G) Concentrations (fmol/cell) of fumarate (B), argininosuccinate (C), citrulline (D), and aspartate (E) were determined in FH1WT and KO MEFs by CE-TOFMS, confirming the metabolic profile observed in equivalent mouse kidneys of significantly elevated fumarate and argininosuccinate and significantly depleted aspartate.

(H) The manganese-containing enzyme arginase catalyzes the conversion of arginine and water to ornithine and urea. Levels of arginine and ornithine (μM) measured in medium by HPLC-MSMS following treatment with bovine arginase show that arginine levels are significantly reduced with time with a concomitant increase in ornithine.

(I–L) WT and KO FH1 MEFs were cultured in medium treated with arginase for 1, 2, 4, and 6 hr compared to no treatment (0 hr). Cells were then returned to standard DMEM (containing arginine) and assayed separately for proliferative capacity (I and J) and colony-forming capacity (K and L).

(I and J) Reduced proliferative capacity of FH1KO MEFs compared to WT controls is observed following culture in arginine-depleted medium for as little as 2 hr. 1.5×10^4 cells/well of 96-well plate were plated for each experimental group and measurements made using CyQuant. Fluorescence units were measured and normalized to those of cells cultured in standard untreated medium.

(K) Representative photographs of colony assays for WT and KO FH1 MEFs 10^3 cells/10 cm plate were cultured for 10 days for each experimental group. Three separate dilutions were plated for each experimental group, and the experiments were repeated in triplicate.

(L) The number of colonies formed by FH1WT and KO MEFs was counted and expressed relative to the colony count of cells cultured in standard untreated medium (0 hr). The graph of plating efficiency versus time cultured in arginine-depleted medium shows that FH1KO MEFs are acutely sensitive to reduced arginine even for a very short time period (2 hr).

See also Figure S2.

Given that FH-deficient cells exhibit a reversal of ASL activity in the urea cycle, we hypothesized that transient arginine depletion might selectively inhibit the growth of FH-deficient cells. Comparison of metabolite concentrations in FH1WT and KO MEFs confirmed the profile observed in equivalent mouse kidneys, of significantly elevated fumarate and argininosuccinate and significantly depleted aspartate (Figures 4D–4G; Table S1). Hence, we cultured FH1KO and WT MEFs for 1, 2, 4, and 6 hr in standard Dulbecco's modified Eagle's medium (DMEM) depleted of arginine by addition of bovine arginase and then returned them to standard DMEM. Since L-arginine is metabolized to L-ornithine and urea by arginase (Shambaugh, 1977), the effectiveness and time course of arginine depletion and parallel increase of ornithine in the medium was confirmed by HPLC-MSMS (Figure 4H). The consequences of arginine depletion for cell proliferation and survival in colony assays were determined and show clearly that culture of FH1KO MEFs for as little as 2 hr in medium depleted of arginine results in loss of proliferative capacity, which increases significantly with time (Figures 4I and 4J). Similarly, there is a significant reduction in the number of FH1KO cells surviving depletion of arginine from the culture medium to form colonies compared to untreated cells or WT control MEFs (Figures 4K and 4L). This further confirms the dependence of FH1KO MEFs on arginine for survival and growth.

DISCUSSION

Here, through global metabolite analyses, we have shown that the urea cycle and arginine biosynthesis are significantly perturbed in FH-deficient cells, cysts, and tumors. Furthermore, arginine biosynthesis occurs predominantly in the cytosol (Salway, 1999) and therefore questions the relative role(s) of mitochondrial and cytosolic FH in oncogenesis. To expand our findings and to assess directly if cytosolic FH has a functional role in vivo, we generated two FH-expressing transgenic mouse lines (including a cytosolic variant, FH^{cyt}), which we have previously demonstrated to suppress both HIF-1α and NRF2 expression in vitro despite a persistent defect in oxidative metabolism (Adam et al., 2011; O'Flaherty et al., 2010). Remarkably, when crossed with an established model of FH-associated cystic disease (Pollard et al., 2007), FH^{cyt} ameliorated cyst development and corrected defects in the urea cycle/arginine metabolism. We hypothesized that FH-deficient cells may be auxotrophic for arginine, a phenomenon often observed in ASS1-negative cancers (Feun et al., 2008). Notably, favorable results have been obtained from clinical trials using pegylated arginine deiminase (ADI-PEG20; Feun and Savaraj, 2006) to treat ASS1-deficient cancers including hepatocellular carcinoma and advanced melanoma (Kuo et al., 2010). We were unable to obtain ADI-PEG20 for this study and therefore used recombinant bovine arginase (Dala and Szajáni, 1994) to deplete arginine acutely in cell culture media. Whereas the viability of FH1KO MEFs was dramatically reduced in comparison to wild-type cells, we did not see the same differential effect between the HLRCC cell lines UOK262 and UOK262+FH (reconstituted). Explanations for this may be that the UOK262 cells are from a distant metastasis and have been propagated extensively in vitro, or that the patient may have received multiple rounds of therapy that have impacted the metabolism of these cells. Generation and analysis of more primary HLRCC and control cell lines are required in order to determine whether arginine depletion would be a suitable therapeutic strategy for HLRCC. Alternatively, other arginase types such as ADI-PEG20 may be more effective in the treatment of human cell lines (Feun and Savaraj, 2006).

The recent identification of succinate dehydrogenase (*SDH*) and *FH* as mitochondrial tumor-suppressor genes has provided a direct link between dysfunctional mitochondria and cancer (King et al., 2006). In contrast, data from our murine models of early stage FH-associated disease indicate a more direct role of fumarate, possibly acting as an oncometabolite (Ternette et al., 2013). This hypothesis has striking parallels with the identification of mutations in another metabolic enzyme IDH1, leading to accumulation of 2-hydroxyglutarate identified as an oncometabolite (Dang et al., 2009; Xu et al., 2011).

Although there are many similarities between FH-deficient MEFs and the UOK262 human cell line, such as lactate production and stabilization of HIF-1α (O'Flaherty et al., 2010; Sudarshan et al., 2009; Yang et al., 2010b), there are also clear differences in their metabolism; for example, the reductive carboxylation pathway is used by UOK262 cells (Mullen et al., 2012) but is not utilized by either FH1KO MEFs or FH1-deficient murine proximal tubular epithelial cells (Frezza et al., 2011b). It is possible that the mouse and human cell lines are models for different stages in the pathogenesis of HLRCC. We would suggest that the FH1 mouse model (both in vivo and in vitro) is a particularly valid model of the early stages of FH deficiency that lead as far as cyst development in vivo, and that factors yet to be defined may then act to drive cells toward neoplasia and further dysregulated metabolism. However, studies using the model have also highlighted pathways that are clearly evident in FH-deficient human tumors, such as succination (Bardella et al., 2011), activation of the NRF2 antioxidant pathway (Adam et al., 2011; Ooi et al., 2011), and, as indicated here, alteration of urea cycle metabolism. The UOK262 cell line perhaps reflects better the later stages of renal neoplasia and metastasis associated with this. UOK262 are quite abnormal cells, and re-expression of either FH or FH^{cyt}, at least in our hands, exacerbates their abnormal morphology and, unlike reconstituted MEFs, does not affect a full "rescue," exemplified by incomplete ablation of normoxic HIF-1α (Figure S2). It is also possible that the UOK262 cells have acquired additional mutations subsequent to loss of FH activity.

The relatively low success rate in treating renal cancer has been attributed in part to high levels of heterogeneity within tumors (Fisher et al., 2012; Gerlinger et al., 2012; Yap et al., 2012), and therefore treatment of HLRCC with arginase could be more effective if explored in combination with other therapies. Thus, tumor and/or metastasis- derived cell lines such as UOK262 will clearly reflect only a subset of the pathological features of such heterogeneous renal cancers.

In summary, we have utilized a murine model of early HLRCC to demonstrate that renal cyst development is independent of mitochondrial FH activity. These studies have highlighted the previously unrecognized importance of the urea cycle and arginine metabolism for FH-deficient cells and tumors and offer a potential Achilles' heel for such cells.

EXPERIMENTAL PROCEDURES

See Extended Experimental Procedures for additional information.

Generation of FH and FH^{cyt} Transgenic Mice

The transgenic constructs consisted of the ubiquitous CAG promoter (Niwa et al., 1991) driving the expression of either full-length (FH) or mitochondria leader sequence deleted (-MLS) (FH^{cyt}) human FH cDNA, both of which carried a V5 tag at the C terminus (Figure 3A), together with a rabbit beta-globin polyadenylation sequence. The two transgenic constructs were cloned into a PhiC31 integrase-mediated exchange vector, pCB92, which was assembled by modifying pExchange4-CB9 (derived from pRMCE (Hitz et al., 2007)) with a synthesized polylinker of unique sites to facilitate cloning. Integration of the constructs occurred specifically at the *ROSA26* locus via PhiC31 integrase-mediated cassette exchange in murine IDG26.10-3 ES cells as previously described (Chen et al., 2011). Correctly integrated ES cell clones were identified by PCR (Figure S1) and injected into mouse C57BL/6J blastocysts, and the resulting chimeric males were mated to C57BL/6 females. Genotypes of transgenic F1 mice were established by PCR (Figure S1). All procedures were conducted in keeping with AACR guidelines and under UK Home Office regulations after approval by the Local Ethical Review process in Oxford University.

Human Tissue Samples

Anonymized human tumor and normal samples were collected with full ethical approval (MREC 05\Q1605\66) as approved by the Oxford Centre for Histopathology Research (OCHRe).

Statistics

All statistical analyses are indicated in the text in the relevant sections. One-way ANOVA and t tests were performed using GraphPad Prism version 5.0d for Macintosh (GraphPad Software, La Jolla, CA, http://www.graphpad.com).

SUPPLEMENTAL INFORMATION

Supplemental Information includes Extended Experimental Procedures, three figures, and one table and can be found with this article online at http://dx.doi.org/10.1016/j.celrep.2013.04.006.

LICENSING INFORMATION

This is an open-access article distributed under the terms of the Creative Commons Attribution License, which permits unrestricted use, distribution, and reproduction in any medium, provided the original author and source are credited.

ACKNOWLEDGMENTS

This work was funded by Cancer Research UK (A13349, A14607, and A12027 to P.J.P.), a Grant-in-Aid for scientific research on Innovative Areas, Japan (no. 22134007 to T.S.), and the Yamagata Prefectural Government and City of Tsuruoka. The Wellcome Trust provided support to P.J.P. (WT091112MA), B.D., D.B., N.S., and the core Microscopy facility at the Wellcome Trust Centre (090532/Z/09/Z). The European Research Council has provided financial support under the European Community's Seventh Framework Programme (FP7/2007-2013)/ERC grant agreement no. 310837 (P.J.P.). T.S. is a founder of Human Metabolome Technologies.

Received: January 15, 2013
Revised: March 24, 2013
Accepted: April 8, 2013
Published: May 2, 2013

REFERENCES

Adam, J., Hatipoglu, E., O'Flaherty, L., Ternette, N., Sahgal, N., Lockstone, H., Baban, D., Nye, E., Stamp, G.W., Wolhuter, K., et al. (2011). Renal cyst formation in Fh1-deficient mice is independent of the Hif/Phd pathway: roles for fumarate in KEAP1 succination and Nrf2 signaling. Cancer Cell *20*, 524–537.

Alderson, N.L., Wang, Y., Blatnik, M., Frizzell, N., Walla, M.D., Lyons, T.J., Alt, N., Carson, J.A., Nagai, R., Thorpe, S.R., and Baynes, J.W. (2006). S-(2-Succinyl)cysteine: a novel chemical modification of tissue proteins by a Krebs cycle intermediate. Arch. Biochem. Biophys. *450*, 1–8.

Ashrafian, H., O'Flaherty, L., Adam, J., Steeples, V., Chung, Y.L., East, P., Vanharanta, S., Lehtonen, H., Nye, E., Hatipoglu, E., et al. (2010). Expression profiling in progressive stages of fumarate-hydratase deficiency: the contribution of metabolic changes to tumorigenesis. Cancer Res. *70*, 9153–9165.

Bardella, C., El-Bahrawy, M., Frizzell, N., Adam, J., Ternette, N., Hatipoglu, E., Howarth, K., O'Flaherty, L., Roberts, I., Turner, G., et al. (2011). Aberrant succination of proteins in fumarate hydratase-deficient mice and HLRCC patients is a robust biomarker of mutation status. J. Pathol. *225*, 4–11.

Bayley, J.P., and Devilee, P. (2010). Warburg tumours and the mechanisms of mitochondrial tumour suppressor genes. Barking up the right tree? Curr. Opin. Genet. Dev. *20*, 324–329.

Chen, C.M., Krohn, J., Bhattacharya, S., and Davies, B. (2011). A comparison of exogenous promoter activity at the ROSA26 locus using a ΦiC31 integrase mediated cassette exchange approach in mouse ES cells. PLoS ONE *6*, e23376.

Dala, E., and Szajáni, B. (1994). Immobilization, characterization, and laboratory-scale application of bovine liver arginase. Appl. Biochem. Biotechnol. *49*, 203–215.

Dang, L., White, D.W., Gross, S., Bennett, B.D., Bittinger, M.A., Driggers, E.M., Fantin, V.R., Jang, H.G., Jin, S., Keenan, M.C., et al. (2009). Cancer-associated IDH1 mutations produce 2-hydroxyglutarate. Nature *462*, 739–744.

Feun, L., and Savaraj, N. (2006). Pegylated arginine deiminase: a novel anticancer enzyme agent. Expert Opin. Investig. Drugs *15*, 815–822.

Feun, L., You, M., Wu, C.J., Kuo, M.T., Wangpaichitr, M., Spector, S., and Savaraj, N. (2008). Arginine deprivation as a targeted therapy for cancer. Curr. Pharm. Des. *14*, 1049–1057.

Feun, L.G., Marini, A., Walker, G., Elgart, G., Moffat, F., Rodgers, S.E., Wu, C.J., You, M., Wangpaichitr, M., Kuo, M.T., et al. (2012). Negative argininosuccinate synthetase expression in melanoma tumours may predict clinical benefit from arginine-depleting therapy with pegylated arginine deiminase. Br. J. Cancer *106*, 1481–1485.

Fisher, R., Larkin, J., and Swanton, C. (2012). Inter and Intratumour heterogeneity: a barrier to individualised medical therapy in renal cell carcinoma? Front. Oncol. *2*, 49.

Frezza, C., Pollard, P.J., and Gottlieb, E. (2011a). Inborn and acquired metabolic defects in cancer. J. Mol. Med. *89*, 213–220.

Frezza, C., Zheng, L., Folger, O., Rajagopalan, K.N., MacKenzie, E.D., Jerby, L., Micaroni, M., Chaneton, B., Adam, J., Hedley, A., et al. (2011b). Haem oxygenase is synthetically lethal with the tumour suppressor fumarate hydratase. Nature *477*, 225–228.

Gerlinger, M., Rowan, A.J., Horswell, S., Larkin, J., Endesfelder, D., Gronroos, E., Martinez, P., Matthews, N., Stewart, A., Tarpey, P., et al. (2012). Intratumor heterogeneity and branched evolution revealed by multiregion sequencing. N. Engl. J. Med. *366*, 883–892.

Hitz, C., Wurst, W., and Kühn, R. (2007). Conditional brain-specific knockdown of MAPK using Cre/loxP regulated RNA interference. Nucleic Acids Res. *35*, e90.

Isaacs, J.S., Jung, Y.J., Mole, D.R., Lee, S., Torres-Cabala, C., Chung, Y.L., Merino, M., Trepel, J., Zbar, B., Toro, J., et al. (2005). HIF overexpression correlates with biallelic loss of fumarate hydratase in renal cancer: novel role of fumarate in regulation of HIF stability. Cancer Cell *8*, 143–153.

Kelly, M.P., Jungbluth, A.A., Wu, B.W., Bomalaski, J., Old, L.J., and Ritter, G. (2012). Arginine deiminase PEG20 inhibits growth of small cell lung cancers lacking expression of argininosuccinate synthetase. Br. J. Cancer *106*, 324–332.

King, A., Selak, M.A., and Gottlieb, E. (2006). Succinate dehydrogenase and fumarate hydratase: linking mitochondrial dysfunction and cancer. Oncogene *25*, 4675–4682.

Kuo, M.T., Savaraj, N., and Feun, L.G. (2010). Targeted cellular metabolism for cancer chemotherapy with recombinant arginine-degrading enzymes. Oncotarget *1*, 246–251.

Loenarz, C., and Schofield, C.J. (2008). Expanding chemical biology of 2-oxoglutarate oxygenases. Nat. Chem. Biol. *4*, 152–156.

Ludwig, C., and Günther, U.L. (2011). MetaboLab—advanced NMR data processing and analysis for metabolomics. BMC Bioinformatics *12*, 366.

Metallo, C.M., Gameiro, P.A., Bell, E.L., Mattaini, K.R., Yang, J., Hiller, K., Jewell, C.M., Johnson, Z.R., Irvine, D.J., Guarente, L., et al. (2012). Reductive glutamine metabolism by IDH1 mediates lipogenesis under hypoxia. Nature *481*, 380–384.

Mullen, A.R., Wheaton, W.W., Jin, E.S., Chen, P.H., Sullivan, L.B., Cheng, T., Yang, Y., Linehan, W.M., Chandel, N.S., and DeBerardinis, R.J. (2012). Reductive carboxylation supports growth in tumour cells with defective mitochondria. Nature *481*, 385–388.

Niwa, H., Yamamura, K., and Miyazaki, J. (1991). Efficient selection for high-expression transfectants with a novel eukaryotic vector. Gene *108*, 193–199.

O'Flaherty, L., Adam, J., Heather, L.C., Zhdanov, A.V., Chung, Y.L., Miranda, M.X., Croft, J., Olpin, S., Clarke, K., Pugh, C.W., et al. (2010). Dysregulation of hypoxia pathways in fumarate hydratase-deficient cells is independent of defective mitochondrial metabolism. Hum. Mol. Genet. *19*, 3844–3851.

Ooi, A., Wong, J.C., Petillo, D., Roossien, D., Perrier-Trudova, V., Whitten, D., Min, B.W., Tan, M.H., Zhang, Z., Yang, X.J., et al. (2011). An antioxidant response phenotype shared between hereditary and sporadic type 2 papillary renal cell carcinoma. Cancer Cell *20*, 511–523.

Pollard, P.J., Brière, J.J., Alam, N.A., Barwell, J., Barclay, E., Wortham, N.C., Hunt, T., Mitchell, M., Olpin, S., Moat, S.J., et al. (2005). Accumulation of Krebs cycle intermediates and over-expression of HIF1alpha in tumours which result from germline FH and SDH mutations. Hum. Mol. Genet. *14*, 2231–2239.

Pollard, P.J., Spencer-Dene, B., Shukla, D., Howarth, K., Nye, E., El-Bahrawy, M., Deheragoda, M., Joannou, M., McDonald, S., Martin, A., et al. (2007). Targeted inactivation of fh1 causes proliferative renal cyst development and activation of the hypoxia pathway. Cancer Cell *11*, 311–319.

Salway, J.G. (1999). Metabolism at a Glance (Malden, MA: Blackwell).

Shambaugh, G.E., 3rd. (1977). Urea biosynthesis I. The urea cycle and relationships to the citric acid cycle. Am. J. Clin. Nutr. *30*, 2083–2087.

Shao, X., Somlo, S., and Igarashi, P. (2002). Epithelial-specific Cre/lox recombination in the developing kidney and genitourinary tract. J. Am. Soc. Nephrol. *13*, 1837–1846.

Soga, T., Igarashi, K., Ito, C., Mizobuchi, K., Zimmermann, H.P., and Tomita, M. (2009). Metabolomic profiling of anionic metabolites by capillary electrophoresis mass spectrometry. Anal. Chem. *81*, 6165–6174.

Sudarshan, S., Sourbier, C., Kong, H.S., Block, K., Valera Romero, V.A., Yang, Y., Galindo, C., Mollapour, M., Scroggins, B., Goode, N., et al. (2009). Fumarate hydratase deficiency in renal cancer induces glycolytic addiction and hypoxia-inducible transcription factor 1alpha stabilization by glucose-dependent generation of reactive oxygen species. Mol. Cell. Biol. *29*, 4080–4090.

Ternette, N., Yang, M., Laroyia, M., Kitagawa, M., O'Flaherty, L., Wolhulter, K., Igarashi, K., Saito, K., Kato, K., Fischer, R., et al. (2013). Inhibition of mitochondrial aconitase by succination in fumarate hydratase deficiency. Cell Rep. *3*, 689–700.

Tomlinson, I.P., Alam, N.A., Rowan, A.J., Barclay, E., Jaeger, E.E., Kelsell, D., Leigh, I., Gorman, P., Lamlum, H., Rahman, S., et al.; Multiple Leiomyoma Consortium. (2002). Germline mutations in FH predispose to dominantly inherited uterine fibroids, skin leiomyomata and papillary renal cell cancer. Nat. Genet. *30*, 406–410.

Warburg, O. (1956). On the origin of cancer cells. Science *123*, 309–314.

Wheatley, D.N. (2005). Arginine deprivation and metabolomics: important aspects of intermediary metabolism in relation to the differential sensitivity of normal and tumour cells. Semin. Cancer Biol. *15*, 247–253.

Wise, D.R., Ward, P.S., Shay, J.E., Cross, J.R., Gruber, J.J., Sachdeva, U.M., Platt, J.M., DeMatteo, R.G., Simon, M.C., and Thompson, C.B. (2011). Hypoxia promotes isocitrate dehydrogenase-dependent carboxylation of α-ketoglutarate to citrate to support cell growth and viability. Proc. Natl. Acad. Sci. USA *108*, 19611–19616.

Xiao, M., Yang, H., Xu, W., Ma, S., Lin, H., Zhu, H., Liu, L., Liu, Y., Yang, C., Xu, Y., et al. (2012). Inhibition of α-KG-dependent histone and DNA demethylases by fumarate and succinate that are accumulated in mutations of FH and SDH tumor suppressors. Genes Dev. *26*, 1326–1338.

Xu, W., Yang, H., Liu, Y., Yang, Y., Wang, P., Kim, S.H., Ito, S., Yang, C., Wang, P., Xiao, M.T., et al. (2011). Oncometabolite 2-hydroxyglutarate is a competitive inhibitor of α-ketoglutarate-dependent dioxygenases. Cancer Cell *19*, 17–30.

Yang, M., Soga, T., Pollard, P.J., and Adam, J. (2012). The emerging role of fumarate as an oncometabolite. Front. Oncol. *2*, 85.

Yang, T.S., Lu, S.N., Chao, Y., Sheen, I.S., Lin, C.C., Wang, T.E., Chen, S.C., Wang, J.H., Liao, L.Y., Thomson, J.A., et al. (2010a). A randomised phase II study of pegylated arginine deiminase (ADI-PEG 20) in Asian advanced hepatocellular carcinoma patients. Br. J. Cancer *103*, 954–960.

Yang, Y., Valera, V.A., Padilla-Nash, H.M., Sourbier, C., Vocke, C.D., Vira, M.A., Abu-Asab, M.S., Bratslavsky, G., Tsokos, M., Merino, M.J., et al. (2010b). UOK 262 cell line, fumarate hydratase deficient (FH-/FH-) hereditary leiomyomatosis renal cell carcinoma: in vitro and in vivo model of an aberrant energy metabolic pathway in human cancer. Cancer Genet. Cytogenet. *196*, 45–55.

Yap, T.A., Gerlinger, M., Futreal, P.A., Pusztai, L., and Swanton, C. (2012). Intratumor heterogeneity: seeing the wood for the trees. Sci. Trans. Med. *4*, 127ps110.

Yogev, O., Naamati, A., and Pines, O. (2011). Fumarase: a paradigm of dual targeting and dual localized functions. FEBS J. *278*, 4230–4242.

Yogev, O., Yogev, O., Singer, E., Shaulian, E., Goldberg, M., Fox, T.D., and Pines, O. (2010). Fumarase: a mitochondrial metabolic enzyme and a cytosolic/nuclear component of the DNA damage response. PLoS Biol. *8*, e1000328.

Zambrowicz, B.P., Imamoto, A., Fiering, S., Herzenberg, L.A., Kerr, W.G., and Soriano, P. (1997). Disruption of overlapping transcripts in the ROSA beta geo 26 gene trap strain leads to widespread expression of beta-galactosidase in mouse embryos and hematopoietic cells. Proc. Natl. Acad. Sci. USA *94*, 3789–3794.

Supplemental Information

Cell PRESS Open ACCESS

EXTENDED EXPERIMENTAL PROCEDURES

Cyst Pathology

Bisected kidneys were fixed in 10% neutral buffered formalin and processed for routine paraffin wax embedding and sectioning (5μm). Hematoxylin and eosin (H&E) stained sections were prepared for all the samples and assessed for pathology as described previously (Adam et al., 2011; Pollard et al., 2007). The frequency and size of renal cysts were determined at different time points for all genotypes in 20 low power (x10) fields with a minimum of n = 8 per experimental group as described before (Adam et al., 2011).

Immunofluoresence

Immunofluorescence combined with confocal microscopy was performed as described previously (O'Flaherty et al., 2010). Cells were labeled first with 250 nM Mitotracker Red CMXRos (Invitrogen), the subcellular localization of V5-tagged FH protein was visualized with V5-FITC conjugated antibody (Invitrogen) and then nuclei were counterstained when the cells were mounted in Vectashield with DAPI (4, 6 diamidino-2-phenyl indole) (Vector labs).

Arginine Depletion from Medium

Arginine-depleted medium was prepared by addition of bovine arginase (MP Biomedicals) (Dala and Szajáni, 1994) to a final concentration of 1U ml^{-1} and 200 μM manganese chloride (Sigma) prior to use for culture of cells (O'Flaherty et al., 2010).

Analysis of Arginine and Ornithine by HPLC-MSMS

Arginine and ornithine were analyzed in methanol extracts using HPLC with tandem mass spectrometric detection. Cell samples (200 μl) were treated initially with 600 μl methanol and the sample centrifuged. An aliquot of the supernatant was further diluted with an equal volume of acetonitrile, centrifuged, and transferred to hplc vials. Separations were carried out using a Waters 2695 separations module (Waters, Elstree, UK) with a Sequant HILIC 3.5 μm, 100 × 2.1 mm hplc column (VWR) maintained at 35°C. The hplc eluents were: A, acetonitrile; B, 50 mM formic acid, with a gradient of 40 – 60% B over 7 min. The flow rate was 0.25 ml min^{-1}, and the injection volume was 2 μl. The amino acids were detected using a Micromass Quattro Micro tandem mass detector in MRM mode using positive electrospray ionization, with the following transitions: ornithine, 133.1 → 70.2, arginine 175.2 → 70.2 and citrulline 176.2 → 70.2. The mass detector employed the following conditions: capillary voltage, 3.00 kV; cone voltage, 16 V; source temperature, 120°C; desolvation temperature, 425°C; desolvation gas flow, 425 l h^{-1}; cone gas flow, 60 l h^{-1}, collision energy 20 V.

Proliferation Assays

Cells were seeded in a 96-well plate with 1.5 x 10^4 cells/well in 100 μl, incubated for at least 1 hr to attach and then the medium was topped up to a final volume of 200 μl/well. Cells in experimental groups were cultured for various time intervals in medium depleted of arginine (as described above). Thereafter medium was removed, the cells washed twice with phosphate buffered saline (PBS) and medium replaced with standard arginine containing medium. The 0 hr time point was measured between 4 and 6 hr after seeding and then at 24 hr intervals for all experimental groups. All measurements were performed in triplicate as follows: medium was replaced with 50 μl CyQuant solution (Life Technologies, Paisley, UK) prepared according to manufacturer's protocol and cells were incubated for 1 hr at 37°C. Plates were read using an EnVision plate reader (PerkinElmer, Cambridge, UK) with an excitation filter at 485/14 nm and an emission filter at 530/10 nm.

Colony Assays

Following treatment with arginine-depleted medium for prescribed time intervals cells were trypsinised and plated in normal medium into five 10 cm plates at 3 cell concentrations (10^2, 5 x 10^2 and 10^3). After 7–10 days colonies were stained with methylene blue (Sigma) (2% in 50% ethanol) and counted using the ColCount (Milton Park, Oxford, UK). Experiments were conducted in triplicate.

Cell Lines and Cell Culture

Four mouse embryonic fibroblasts (MEFs) cell lines were used: FH1fl/fl (FH1WT), FH1−/− (FH1KO), and isogenic FH1KO MEFs reconstituted with either full-length FH (FH1KO+FH), or cytosolic-restricted FH by deleting the mitochondrial targeting sequence (FH1KO+FH^{cyt}) as described in (O'Flaherty et al., 2010). UOK262 cells (Yang et al., 2010b) were transfected using FuGene6 (Roche) with either full length human FH (FH), or FH without the mitochondrial targeting sequence (FH^{cyt}), following the manufacturer's protocol. Following G418 selection, stable clones were expanded and analyzed by immunofluorescence to confirm subcellular localization of the transcripts via the C-terminal encoded V5 tag, as described previously (O'Flaherty et al., 2010). All cell lines were cultured in DMEM (PAA Laboratories) containing 4500 mg L^{-1} glucose), 10% fetal calf serum (Sigma), and 2 mM glutamine (Sigma).

Immunoblotting

Immunoblotting was performed as described in (O'Flaherty et al., 2010). Antibodies used were: FH (Autogen Bioclear), V5 (Invitrogen), HIF-1alpha (Cayman labs) and ACTB-HRP (Abcam).

Glutamine-2,3,3,4,4-d5 Labeling of Cells and CE-TOFMS

Cells were cultured in 10 cm plates in standard DMEM for 12 hr. The medium was replaced with medium containing 4 mM [D5]-Glutamine (Glutamine-2,3,3,4,4-d5) for 3 or 9 hr. Cells were then processed for isotope incorporation and metabolite analysis. In all CE-TOFMS experiments we used the Agilent CE capillary electrophoresis system (Agilent Technologies, Waldbronn, Germany), the Agilent G3250AA LC/MSD TOF system (Agilent Technologies, Palo Alto, CA). For anionic metabolite profiling, the original Agilent stainless steel ESI needle was replaced with the Agilent G7100-60041 platinum needle (Soga et al., 2009). Cationic metabolites were separated in a fused silica capillary (50 μm i.d. x 100 cm) filled with 1 M formic acid as the electrolyte (Soga et al., 2003). A sample was injected at 50 mbar for 3 s (3 nL), and 30 kV voltage was applied. Methanol-water (50% v/v) containing 0.1 μM Hexakis (2,2-difluorothoxy) phosphazene was delivered as the sheath liquid at 10 μl min^{-1}. ESI-TOFMS was in the positive ion mode, and the capillary voltage was set at 4 kV. The flow rate of heated dry nitrogen gas (heater temperature 300°C) was maintained at 10 psig. At TOFMS, the fragmentor-, skimmer- and Oct RFV voltages were set at 75-, 50-, and 125 V, respectively. Automatic recalibration of each acquired spectrum was with reference masses of reference standards ([^{13}C isotopic ion of protonated methanol dimer ($2MeOH^{+}H$)]$^{+}$, m/z 66.0632) and ([Hexakis (2,2- difluorothoxy)phosphazene +H] $^{+}$, m/z 622.0290). Exact mass data were acquired at a rate of 1.5 spectra s^{-1} over a 50 - 1,000 m/z range (Soga et al., 2006). For anionic metabolites, a cationic polymer-coated COSMO(+) capillary (50 μm i.d. x 110 cm) (Nacalai Tesque, Kyoto, Japan) was used as the separation capillary. A sample (30 nL) was injected at 50 mbar for 30 s, and −30kV of voltage applied. 5 mM ammonium acetate in 50% (v/v) methanol-water containing 0.1 μM Hexakis was delivered as the sheath liquid at 10 μl min^{-1}. ESI-TOFMS was conducted in the negative ionization and the capillary voltage was set at 3,500V. Other conditions are described previously (Soga et al., 2009).

$^{15}N_4$,$^{13}C_6$-Arginine Labeling of Cells

Cells were cultured in 10 cm plates in standard DMEM for 12 hr. The medium was then replaced by fresh medium supplemented with 0.4 mM $^{15}N_4$,$^{13}C_6$-arginine for 6 or 12 hr. Media and cell lines were then processed for metabolomics analyses. Extraction and analysis by CE-TOFMS was performed as described briefly below and previously (Soga et al., 2006; Soga et al., 2009; Sugimoto et al., 2005).

Heteronuclear Single Quantum Correlation Nuclear Magnetic Resonance Analysis

2D-^{1}H,^{13}C-HSQC NMR spectra were collected on a 600 MHz Bruker Avance III spectrometer (Bruker Biospin, UK) with a TCI 1.7 mm z-PFG cryogenic probe at 300K. The spectral widths were set to 7812.5 and 24146.38 Hz for the ^{1}H and ^{13}C dimension. A total of 2048 complex data points was acquired for both dimensions to resolve small chemical shift differences between arginine and argininosuccinate and to resolve the ^{13}C-^{13}C couplings in the indirect dimension. Two transients were acquired after 8 steady-state scans, the interscan relaxation delay was set to 2 s. The NMR spectra were processed using the MATLAB-based NMRLab/MetaboLab software (Ludwig and Günther, 2011). Each spectrum was zero-filled to 4k data points in both dimensions prior to Fourier Transformation. The chemical shift was referenced to the methyl group of alanine based on its assignment in the human metabolome database (Wishart et al., 2007).

Metabolite Analysis of Kidney Samples

Kidneys were harvested, bisected and frozen immediately. The frozen samples were then homogenized without thawing by a cell disrupter (MS-100R; TOMY, Tokyo, Japan) at 2°C following the addition of 500 μl methanol containing 20 μM of each of the internal standards Methionine sulfone (MetSul) (Avocado) and 2-(N-morpholino)-ethanesulfonic acid (MES) (Dojindo). The homogenate was mixed with 200 μl Milli-Q water and 500 μl chloroform and centrifuged at 9100*g* at 4°C for 4 hr. The resulting aqueous layer was filtered centrifugally through a 5 kDa cut-off filter (Millipore) to remove proteins. The filtrate was concentrated centrifugally and dissolved in 50 μl Milli-Q water containing 200 μM each of the reference compounds 3-Aminopyrrolidine (Aldrich) and 1,3,5- Benzenetricarboxylic acid (Trimesate) (Wako). The sample solution was further diluted with Milli-Q water prior to CE-TOFMS and the concentration of each metabolite was calculated as described previously (Soga et al., 2009).

Metabolite Analysis of Cell Samples

All cells are adherent. When cells were harvested for CE-TOFMS the medium was aspirated and the cells washed twice in an excess of 5% Mannitol (Wako). Methanol containing 3 standards (MetSul, MES and D-Camphor-10-sulfonic acid (CSA) (Wako) each at 25 μM) was added. This was left at rest for 10 min and sample solution harvested. Samples were processed for CE-TOFMS essentially as described above and as published previously (Soga et al., 2009).

SUPPLEMENTAL REFERENCES

Soga, T., Baran, R., Suematsu, M., Ueno, Y., Ikeda, S., Sakurakawa, T., Kakazu, Y., Ishikawa, T., Robert, M., Nishioka, T., and Tomita, M. (2006). Differential metabolomics reveals ophthalmic acid as an oxidative stress biomarker indicating hepatic glutathione consumption. J. Biol. Chem. *281*, 16768–16776.

Soga, T., Ohashi, Y., Ueno, Y., Naraoka, H., Tomita, M., and Nishioka, T. (2003). Quantitative metabolome analysis using capillary electrophoresis mass spectrometry. J. Proteome Res. *2*, 488–494.

Sugimoto, M., Kikuchi, S., Arita, M., Soga, T., Nishioka, T., and Tomita, M. (2005). Large-scale prediction of cationic metabolite identity and migration time in capillary electrophoresis mass spectrometry using artificial neural networks. Anal. Chem. *77*, 78–84.

Wishart, D.S., Tzur, D., Knox, C., Eisner, R., Guo, A.C., Young, N., Cheng, D., Jewell, K., Arndt, D., Sawhney, S., et al. (2007). HMDB: the Human Metabolome Database. Nucleic Acids Res. *35*(Databaseissue), D521–D526.

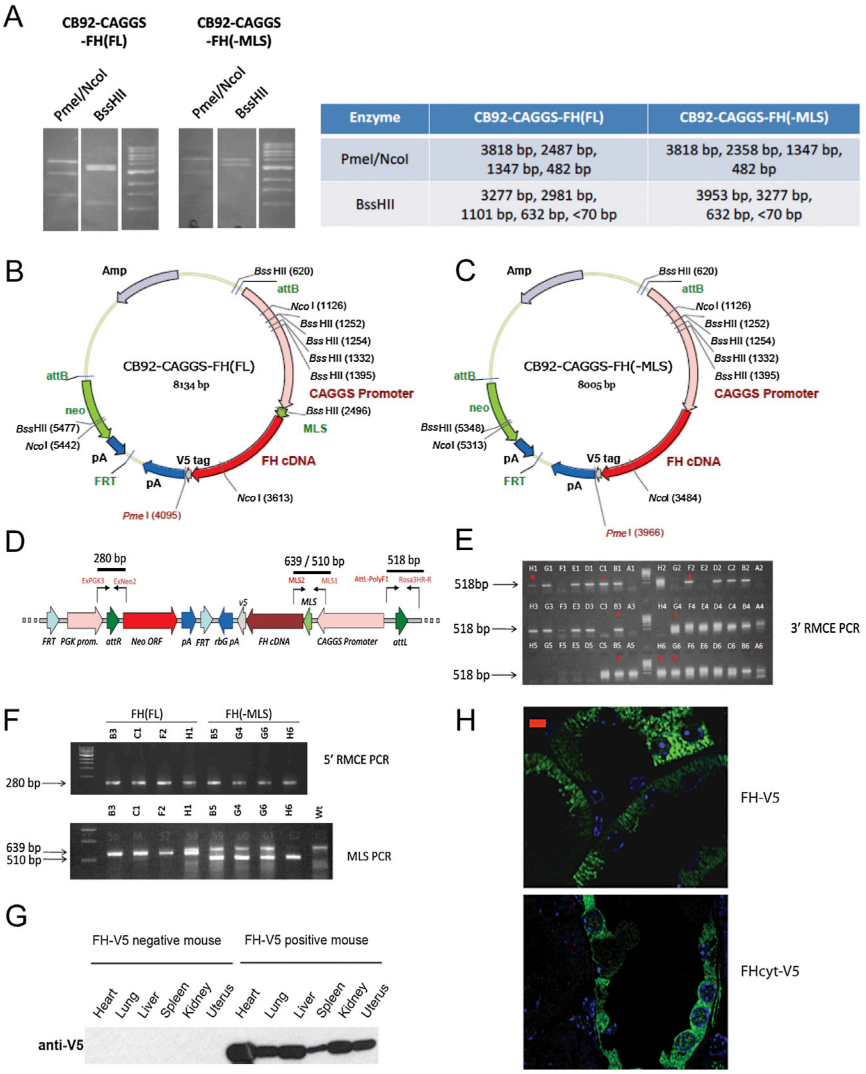

Figure S1. Generation and Analyses of FH (FL) (FH) and FH(-MLS) (FH^{cyt})-Expressing Transgenic Mice, Related to Figure 2

(A) Restriction digest profile of CB92-CAGGS-FH(FL) (FH) and CB92-CAGGS-FH(-MLS) (FH^{cyt}), showing the predicted fragments. (MLS = mitochondrial leader sequence).

(B and C) show the plasmid maps of the two exchange vectors, including the sequenced regions and the locations of the restriction sites used for the restriction analysis.

(D) The exchanged *Rosa26* loci (Chen et al., 2011) are shown with the positions of the genotyping primers.

(E) PCR genotyping for exchange at the 3′ end using primer combination AttL-PolyF1 and Rosa3HR-R (Clones ending in 1, 2 or 3 contain the full length construct and clones ended in 4, 5 or 6 contain the MLS deleted construct).

(F) PCR genotyping for exchange at the 5′ end using primer combination ExPGK3 and ExNeo2 (top panel) and PCR genotyping for the presence of the transgenic construct allowing the two variant (full length and MLS deleted) to be distinguished on the basis of size (bottom panel).

(G) Verification by immunoblot of the presence of FH-V5 in tissues of transgenic mice. Such verification was confirmed in tissues from three mice for both FH(FL) (FH) and FH(-MLS) (FH^{cyt}).

(H) Confirmation by immunofluorescence of the localization of FH-V5 and FH^{cyt}-V5 in renal tubular cells. Scale bar = 5 µm.

Cell PRESS
Open ACCESS

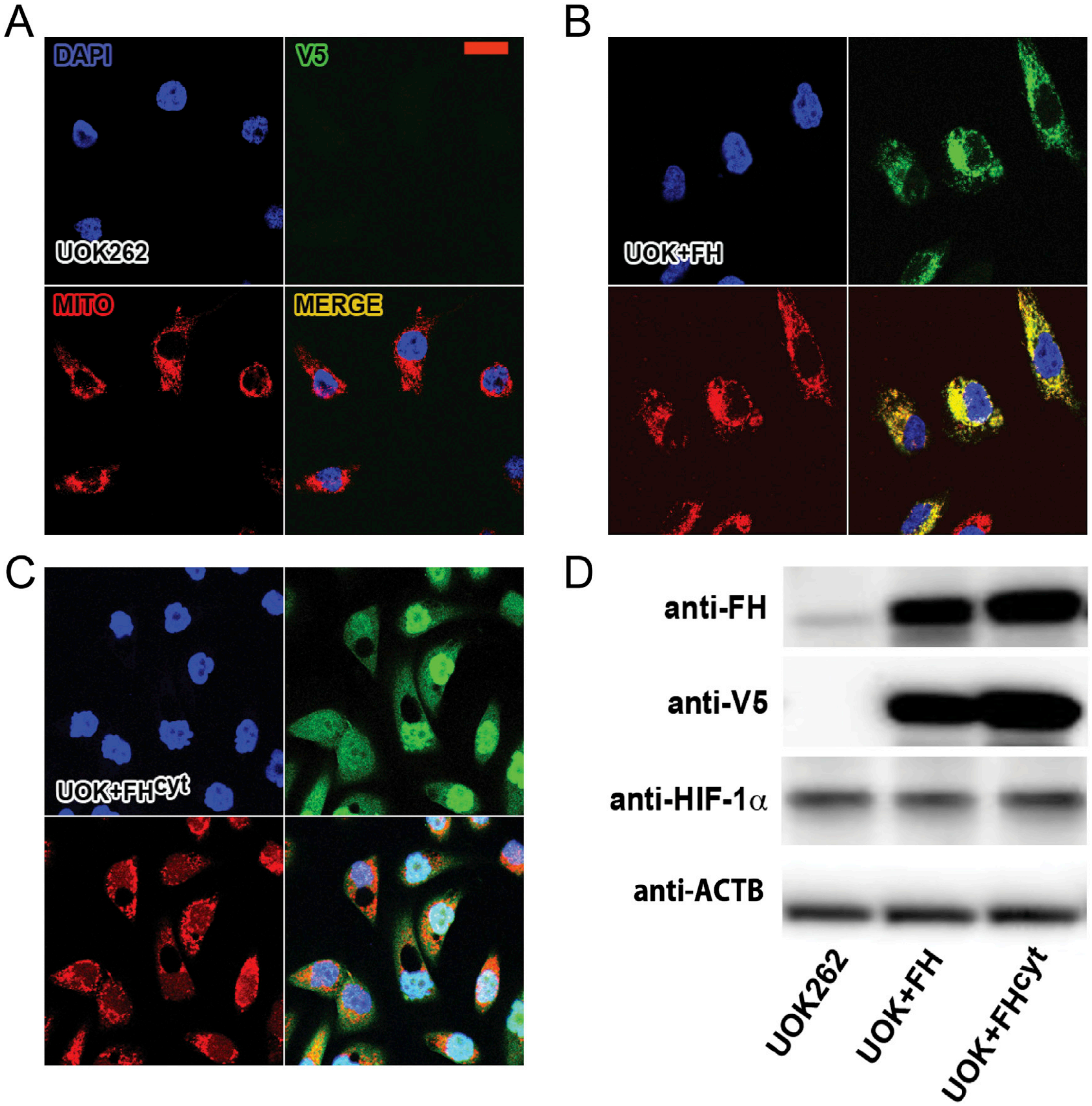

Figure S2. Characterization of UOK262 Cells Complemented with FH and FH^{cyt}, Related to Figure 4

(A-C) Complementation with, and localization of, FH and FH^{cyt} in UOK262 cells (Yang et al., 2010b) was confirmed by immunofluorescence. The FH and FH^{cyt} constructs carry a C-terminal V5 tag (green); mitochondria are identified with Mitotracker red (red) and nuclei by Dapi (blue).

(A) Parental UOK262 cells show no V5 immunofluorescence. Four panels are shown; three for each fluorophore separately and a merged image.

(B) UOK262 cells transfected with FH show clear overlap with Mitotracker red confirming the mitochondrial localization.

(C) Cytoplasmic localization of FH^{cyt} was confirmed since there is no overlap with Mitotracker red. The large vacuoles that are present in these cells can also be seen clearly in this set of images.

(D) Complementation of UOK262 cells with FH and FH^{cyt} was confirmed further by Western blot analysis for FH and the C-terminal V5 tag. ACTB was used as a loading control. Note that complementation with either transgene did not ameliorate HIF-1α levels.
